# May Exercise Prevent Addiction?

**DOI:** 10.2174/157015911795017380

**Published:** 2011-03

**Authors:** C. A Fontes-Ribeiro, E Marques, F. C Pereira, A. P Silva, T. R. A Macedo

**Affiliations:** Institute of Pharmacology and Experimental Therapeutics, Biomedical Institute for Research on Light and Image (IBILI), Faculty of Medicine; Association for Biomedical Research and Innovation on Light and Image (AIBILI); 3000 Coimbra, Portugal

**Keywords:** Amphetamine, Conditioned-place-preference, Exercise, Training, Addiction.

## Abstract

Amphetamines exert their persistent addictive effects by activating brain's reward pathways, perhaps through the release of dopamine in the nucleus accumbens (and/or in other places). On the other hand, there is a relationship between dopamine and all behavioural aspects that involve motor activity and it has been demonstrated that exercise leads to an increase in the synthesis and release of dopamine, stimulates neuroplasticity and promotes feelings of well-being. Moreover, exercise and drugs of abuse activate overlapping neural systems. Thus, our aim was to study the influence of chronic exercise in the mechanism of addiction using an amphetamine-induced conditioned-place-preference in rats.

Adult male Sprague-Dawley rats were randomly separated in groups with and without chronic exercise. Chronic exercise consisted in a 8 week treadmill running program, with increasing intensity. The conditioned place preference test was performed in both groups using a procedure and apparatus previously established. A 2 mg.kg^-1^ amphetamine or saline solution was administered intraperitonially according to the schedule of the conditioned place preference.

Before conditioning none of the animals showed preference for a specific compartment of the apparatus. The used amphetamine dose in the conditioning phase was able to produce a marked preference towards the drug-associated compartment in the group without exercise. In the animals with exercise a significant preference by the compartment associated with saline was observed.

These results lead us to conclude that a previous practice of regular physical activity may help preventing amphetamine addiction in the conditions used in this test.

## INTRODUCTION

Drug addiction is characterized by a sequence of processes that lead to the relapsing nature of the disorder. Since the dopaminergic mesocorticolimbic system is considered to be involved in reward-related associative learning [1], reinforcement [2] and incentive salience [3], it may have a crucial role in the development of drug addiction. On the other hand, physical activity involves the synthesis and release of dopamine in the basal ganglia [4]. Therefore, the dopaminergic mesocorticolimbic brain system seems particularly involved in both the execution of physical activity and in some characteristics that may lead to the development of drug addiction.

Although one of the main public health goals is maintaining brain health and plasticity throughout life, it is becoming clear that physical activity and behavioural stimulation can help us to achieve such objective, since they can act as a powerful trigger not only for peripheral adaptive processes, such as cardiovascular and musculoskeletal adaptations, but also for brain plasticity [5-12]. Some of these beneficial effects are observed in diseases such as depression [13] and Parkinson’s disease [14-16], and even some neurorestoration properties have been recognized in studies using basal ganglia injuries [17].

There are two types of methods to evaluate drug addiction: 1. The self-administration models (Operant and Non-Operant), that are widely used in basic drug abuse research, because they are well structured and show face validity towards drug consumption in humans; 2. Models where the drug is administered by the experimenter at a dosage and temporal distribution independent of the subject’s will and behaviour. One example of the latter is the conditioned place preference (CPP) which was introduced in the 1980s to compensate methodological and interpretive difficulties associated with the self-administration technique. Although CPP measures a learning process fundamentally distinct from drug self-administration, it presents main advantages: (1) the possibility to test animals in a drug-free state; (2) sensitivity to motivational properties such as rewarding or aversive drug effects; (3) the possibility to use CPP and determine locomotor activity simultaneously; (4) the possibility to be applied to several species. It is also important to refer the existence of some limitations that include: (1) the interference of the notion of “novelty seeking” in the interpretation of results; (2) the difficulty to obtain graded dose-effect curves needed in some pharmacological studies; (3) the possibility of a previous drug-conditioning context preference demonstrated by animals; and (4) the yet not demonstrated validity as an experimental protocol of drug reward in humans.

Despite the above limitations, the CPP paradigm is widely accepted as a behavioural approach to better understand the neural mechanisms of rewarding effect and for screening drugs for abuse liability [18].

Thus, taking into account that both drug addiction and physical activity share the dopaminergic system, our attention in this study was focused in the influence of chronic exercise in the prevention of addiction using an amphetamine-induced conditioned-place-preference in rats, a popular model of drug-mediated associative learning.

## MATERIAL AND METHODS

Adult male Sprague-Dawley rats (Charles River Laboratories Inc., Barcelona, Spain), 8 weeks old, were housed (3 per cage) under controlled conditions of room temperature (21 ± 1ºC), humidity, and with light/dark cycles of 12 h, supplied with food and water *ad libitum*. 

They were randomly separated in a group that performed chronic exercise (“With Training”), and in other group that did not practice exercise (“Without Training”). Chronic exercise consisted in a 8 weeks treadmill running program (Panlab/Letica LE8706, Barcelona, Spain), with increasing total session time (from 15 min in the beginning to 50 min in the last week) and intensity (daily maximal velocity increased from 12 cm/s to 54 cm/s and a 5% slope included in the fourth week).

In order to perform the conditioned place preference, animals were moved one hour earlier to the test room, being kept in the dark (with a dim red light allowing only the operator to see) and with minimal noise interference, in order to minimize stress in the animals. The test was performed in an apparatus consisting in two principal compartments (63x30x30 cm), connected by a smaller one (63x10x10 cm) (Panlab/Letica LE8706, Barcelona, Spain). The principal compartments have two different colours (white and black) and floor textures (rough and smooth, respectively) to be perfectly distinguished by the animals and to prevent any innate preference. This apparatus was previously tested.

This CPP test consists in three phases. The first one is the pre-conditioning phase, when animals are allowed to explore all the compartments of the apparatus for 20 min and the time spent in each compartment registered, in order to test the animals for any innate preference for a specific compartment. In the second phase, or conditioning phase, animals from both groups (with and without training) are randomly selected to the saline or amphetamine treated group. This phase has the duration of 8 consecutive days and animals were injected intraperitonially with 2 mg.kg^-1^ amphetamine and saline solution (NaCl 0.9%) in every other day (total of 4 injections of amphetamine or saline solution), being the compartments associated to one of the conditions. We used an unbiased conditioned place preference protocol, being the drug injection associated with an arbitrarily chosen compartment, counterbalanced across individuals. As the rewarding effect has a temporal profile that presumably increases and then decreases across time since administration, animals were injected and immediately placed in the previously chosen compartment for 45 min. The third phase (post-conditioning or test phase) is similar to the pre-conditioning one (free access to compartments for 20 min, and registration of the time spent in each of them).

All the experiments were performed under the rules of the European Convention on Animal Care. 

## STATISTICAL ANALYSIS

Results are presented in time (seconds), and data are shown as mean ± standard error of the mean (SEM). The differences between means within groups comprising trained and untrained animals were analysed by unpaired two-tailed student´s t-test. Significant differences were defined at p < 0.05. 

## RESULTS

**Fig. (1):**
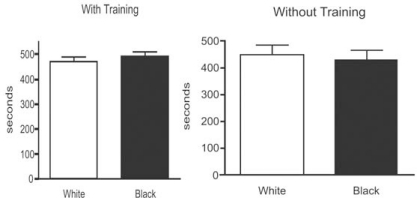
Time (in sec) spent in each compartment (black with smooth floor or white with rough floor) in the Pre-Conditioning Phase, for trained and untrained groups. Results shown are mean ± SEM; n=6-10 rats per group.

In the first phase of the conditioned place preference test (pre-conditioning phase), animals were tested in a drug-free state and both groups (with and without training) spent similar times exploring the apparatus (Fig. **1**). Animals that had a previous training presented no significant differences between mean time spent in the white compartment with the rough floor (470.3 ± 17.5 sec, n = 16) and in the black compartment with the smooth floor (479.6 ± 16.6 sec, n = 16). The animals without training presented similar results, spending no significant different mean times (p > 0.05) in the white compartment (447.5 ± 37.9 sec, n = 14) and in the black compartment (428.4 ± 36.0 sec, n = 14). 

As in this pre-conditioning phase none of the animals presented preference for a specific compartment, all of them were used in the next phase of the test (conditioning phase). 

**Fig. (2):**
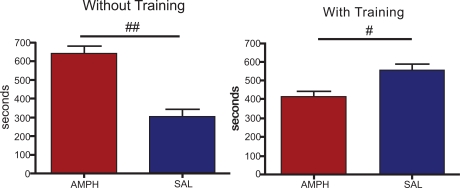
Time (in sec) spent in compartments paired with saline or amphetamine (2 mg.kg^-1^) after conditioning, for trained and untrained groups. Results shown are mean ± SEM; n=6-10 rats per group. # p < 0.05; ## p < 0.01.

After conditioning, the group without chronic exercise showed a marked preference for the compartment previously associated with amphetamine (Fig. **2**). The mean time spent by these animals in the amphetamine associated compartment (641.9 ± 40.9 sec, n = 8) is significantly different from the mean time spent in the compartment where the saline solution was administered (304.5 ± 36.2 sec, n = 8). 

On the other hand, animals with chronic exercise presented a significant higher mean time spent in the compartment associated with the saline injection (556.3 + 34.7 sec, n = 8) than in that associated with the amphetamine compartment (416.6 + 27.5 sec, n=8) (Fig. **2**).

## DISCUSSION

Over a decade ago, a theory proposed that there could be a common brain circuitry underlying both drug reward and locomotor’s stimulation [19]. Our results seem to suggest that exercise by recrutiting this common brain pathway prevent amphetamine-seeking behaviour.

The used training protocol was designed considering that moderate regular exercise for at least six weeks can bring beneficial effects in a long term basis, not only at cardiovascular and muscular, but also at a neurochemical level [20, 21]. We have to consider that this type of exercise is forced and not spontaneous. Therefore, one can not rule out the contribution played by stress inherent to the training procedure to the obtained results [22]. In fact, previous studies show that activation of prefrontal circuits may play a crucial role in mechanisms by which some kind of stress stimulates reward circuits [23-25]. 

Although presenting some disadvantages, the conditioned place preference, as previously mentioned, has been successfully used in measuring the reward effect of drugs like amphetamines in rats [18, 26].

One of the mentioned problems in analyzing the results obtained could be the presence of an innate preference for one of the compartments. To avoid this situation, a previous test was performed with a small number of animal (results not shown) used to calibrate the apparatus. In this previous test we could verify a preference for the black compartment before introducing a difference in the compartments floor textures. As mentioned by Vezina and Stewart [27], a single element can control a place preference as a Pavlovian behaviour, and we could revert what seemed an innate preference for that particular compartment after introducing a different tactile stimuli from texture flooring. With this decision, any strong bias in preference was minimized, as shown in the pre-conditioning results. Thus, an unbiased procedure was used in the conditioning phase.

The problem mentioned as novelty seeking behaviour, that could interfere with the obtained results analysis, was solved by the use of a third compartment, as suggested by Mucha and Iversen [28] and Parker [29]. The novel environment is presented to all animals and the time spent in the drug-paired compartment is superior to the time spent in the novel environment. In accordance to the expected, the group without training spent a significant longer time in the drug-paired compartment. Conversely, the group with training spent more time in the compartment associated with the saline administration.

Another major problem is due to the fact that the dose-effect results are not manifested until the final test day, following conditioning, thus preventing adjustments in the tested doses as the experiment progresses. Once the group without training after conditioning showed a marked preference by the drug paired compartment, we can assume that the chosen drug administration protocol (4 x 2 mg.kg^-1^ amphetamine; one injection every other day) is able to induce the drug-seeking behaviour. In summary, since all the major problems associated to the conditioned place preference apparatus were controlled, we can confirm the efficacy of the test and so the obtained results can be reliable.

Considering that the conditioned place preference test under the selected conditions (amphetamine dose, floor textures, general room conditions) shows a drug-seeking behaviour in the untrained animals, the significant higher mean time spent in the saline associated compartment by animals with training represents a positive effect of chronic exercise in drug rewarding properties. Therefore, we can hypothesize that a moderate to high intensity regular exercise is altering the reward circuitry/mechanisms for amphetamine (in this dose). To complement this fact, other groups already notice beneficial effects of training procedures in those detrimental/harmful effects of drug consumption, like impairment in short-term memory and learning [30]. According to this information we can view that our results are in accordance to previous studies, bringing forward the idea that a training protocol consisting in regular exercise, maintained for a long period of time, may abolish the drug-seeking behaviour, one of the steps that can lead to drug addiction.

Although exercise may prevent drug addiction, one should be aware that when the exercise schedules interfere with the social, occupational and family lives it can be consider itself as a dependence [31].
